# A High-Performance Transmitarray Antenna with Thin Metasurface for 5G Communication Based on PSO (Particle Swarm Optimization)

**DOI:** 10.3390/s20164460

**Published:** 2020-08-10

**Authors:** Chengtian Song, Lizhi Pan, Yonghui Jiao, Jianguang Jia

**Affiliations:** 1 School of Mechatronical Engineering, Beijing Institute of Technology, Beijing 100081, China; 3220180119@bit.edu.cn (L.P.); 3220190122@bit.edu.cn (Y.J.); 2 Institute of Systems Engineering, AMS, PLA, Beijing 100091, China; jiajianguang@126.com

**Keywords:** high gain, transmitarray (TA) antenna, metasurface (MS), PSO, side-lobe level (SLL) reduction

## Abstract

A 5G metasurface (MS) transmitarray (TA) feed by compact-antenna array with the performance of high gain and side-lobe level (SLL) reduction is presented. The proposed MS has two identical metallic layers etched on both sides of the dielectric substrate and four fixed vias connecting two metallic layers that works at 28 GHz to increase the transmission phase shift range. The proposed planar TA consisting of unit cells with different dimensional information can simulate the function as an optical lens according to the Fermat’s principle, so the quasi-spherical wave emitted by the compact Potter horn antenna at the virtual focal point will transform to the quasi-plane wave by the phase-adjustments. Then, the particle swarm optimization (PSO) is introduced to optimize the phase distribution on the TA to decrease the SLL further. It is found that the optimized TA could achieve 27 dB gain at 28 GHz, 11.8% 3 dB gain bandwidth, −30 dB SLL, and aperture efficiency of 23% at the operating bandwidth of 27.5–29.5 GHz, which performs better than the nonoptimized one. The advanced particularities of this optimized TA including low cost, low profile, and easy to configure make it great potential in paving the way to 5G communication and radar system.

## 1. Introduction

Followed by the increasing demands of high-speed data switching rate, the fourth-generation wireless transmission protocol is not enough for the demanding data usage in many areas as more and more devices are connected in the narrow channels exactly like the traffic jam. To handle this problem, the next-generation system, 5G, is developing with a better performance in capacity, information transmitted, and energy efficiency, and showing great prospects for applications [1,2,3,4]. Allocated by the Third Generation Partnership Project (3GPP), the spectrum available for 5G network is subdivided into lower frequency part and higher frequency part which is made 6 GHz as the separation [1,2]. Although compared with the narrow channels and the higher loss in sub-6 GHz bands, the higher frequency band such as 66 and 28 GHz, with the general properties of lower path loss caused by the atmospheric absorption and less occupied channels, will be a competitive candidate for the development of 5G communication [3,4], and the research on the array antenna are getting more and more attention in recent years to satisfy the stricter requirements of 5G.

Historically, in the field of the international communication industry, the array antenna plays an irreplaceable role since it was invented. Owning to the performance of high gain, low side lobe level (SLL), and wide bandwidth, the array antenna always requires different forms to satisfy the growing corresponding needs [5,6,7,8,9,10,11,12,13,14]. The phased-array (PA) antenna performs various functions in the communication system [5,6,7,8]. It has been demonstrated that the PA has several advantages, such as compact array and high gain [5,6,7,8]. To circumvent the complexity of feeding networks, single microstrip antennas for high-directivity have been proposed [9]. PA, however, suffers from the complicated feeding networks and complex design of the excited phase at different ports.

To simplify the design and further enhance the gain, thin planar array with separated feed antenna, such as the reflectarray (RA) antenna and transmitarray (TA) antenna, are proposed in the areas of long-distance communication and radar system [10,14,15,16]. As for the RA, the metal ground usually covers one side of the array to reflect the incident wave, so only the single layer with almost 0 dB (close to 1) return loss is required, and only the phase distribution is needed to be considered in the design without the worry of the amplitude distribution. The radiation mechanism and a general design method of the RA is discussed in detail in Ref. [10]. In Ref. [11], the patches with identical phase-delay lines but different element rotation angles were used to regulate the reflected phase distribution to obtain high gain and beam scanning. Regardless of the advantages of low return loss and single layer, RA also faces some constraints, like the patch feed and the horn feed have the blocking effect to the reflected wave, which will enhance the backward radiation of RA [12,13]. In Ref. [17], ultrawideband low-profile TA feed by Vivaldi array, which has relatively large size, is discussed. 

To overcome these shortages, the TA is developed to overcome the shortcoming of the RA and has greater tolerance to the surface errors [18]. Unlike the incident wave and reflected wave at the same side of the RA, the incident wave illuminates on the TA and passes through the array becoming the transmitted wave, so the blocking effect could be avoided largely. In general, there are mainly two ways to classify the TA. One is according to the number of layers of the dielectrics; the four-layer structures are presented in [15,19,20,21,22] and three-layer structure is proposed in the Ref. [23], while the two-layer structures are exploited, respectively. The other is based on the various working principles, TA could be further categorized into four different types, namely, frequency selective surfaces (FSS) [15,18,19,20,22,24], metasurface (MS) [21,23,25,26], the spatial bandpass-filter based structure [27], receive/transmit-mode structure, and so on [16,28,29]. Owing to the transmission, amplitude and phase should be taken into consideration at the same time, how to simply the TA while keeping the specific properties of lower SLL as well as greater surface tolerance for the application in 5G communication has become an urgent research hotpot. The method of optimization can be divided into two categories: local and global search algorithms [30]. Literally, the local optimization is worked in a limited space with designed initial values, whereas the global optimization is forced on the entire solution hyperspace and just the random initial values are needed and the best result will be found in the whole search space [31]. As for the optimization of phase distribution, there are always hundreds of elements that need to be optimized and it is difficult to set up suitable initial values for all the elements befittingly, so the global search algorithms are used here for the optimization of phase distribution. Considering the flexible and independent properties of the elements on the array, heuristic method is widely adopted in the optimization of array antenna among which, genetic algorithm (GA) and particle swarm optimization (PSO) show great superiority at the aspect of efficiency and operability [31,32,33,34,35,36]. Though PSO and GA both belong to the population-based stochastic optimization, PSO has only two lines of kernel codes compared to the recurrent process of crossover, breeding, and selection in GA. PSO also benefits from less computational bookkeeping with less computational resource [33,34].

In this paper, we propose a two-layer MS TA antenna feed by the optimized horn antenna to actualize the reduced SLL and high gain at the frequency range around 28 GHz for the 5G communication. This thin TA comprises a single substrate and two circular patches on both sides of the substrate, and four metal vias are inserted into the substrate at the centrally symmetrical positions for each element, which is based on structure presented in the Ref. [28] working at 20 GHz but with more simple and efficient design. The positions of vias are fixed at the changeless positions on the array in this paper, while the positions of vias changed with the varying patch size to match the requirements of phase distribution in the published paper. Only two sizes of circular patches are used, and the length of slots on the patches will control the transmitted phase. To the author’s knowledge, it is the first time when the transmission coefficients are controlled using this structure. Hence, a TA structure with lower cost and easier fabrication is presented in this paper. Then, an optimized Potter horn antenna is exploited as feed structure of the TA, which has good symmetry in principal plane patterns as the corrugated horns, and the smooth wall of the Potter horn greatly reduces the difficulty of manufacturing. The TA and the Potter horn antenna make the whole prototype as a 5G TA antenna, and a 27 dBi maximum gain in the main direction is achieved, while the 3-dB gain bandwidth and aperture efficiency are maintained at 11.8% and 28%, respectively. The SLL is maintained at −20 dB level and after the optimization, the SLL can achieve the level of −30 dB, which can be used as the high-gain array antenna for 5G directional communication. Our contributions are listed as follows: in Section 2, first, a horn antenna is optimized by cos^q^ (θ)-mode method to get the approximate uniform radiation in azimuthal direction; second, a transmission unit cell with single substrate layer was designed in an easier way without using multisubstrate layer structure or air gaps; and third, particle swarm optimization is used to optimize the SLL of TA antenna radiation pattern. In Section 3, the optimized results are compared with the published paper and show better performance in the SLL decreasing. 

## 2. Transmitarray Antenna Design

In the design of thin planar array antenna with separated feed, there are always five steps [37]. The first step is to find a suitable feed structure. The feed at the optimized position is an important factor related to the gain enhancement and the SLL reduction. The following step is the analysis of unit cell. Periodical boundary condition with plane wave incidence are used to get series of transmission coefficients by sweeping the related dimension. The third step will be the design of TA according to the transmission coefficients at 28 GHz, which are extracted from the previous step. In addition, the fourth step will be the joint simulation of TA and feed structure to achieve the desired performance, furthermore, the final step is to optimize the transmission coefficients of elements at different positions on the array to reduce the SLL, which will be discussed in the next section.

### 2.1. Feed Antenna Design

Typically, patch antenna and a horn antenna are often used as the feed source of TA and RA [12,13,14,15,18,19,20,21,22,23,26,27,28,38]. The substrate integrated waveguide (SIW) antenna is often used as the feed of MIMO TA antenna for the beam forming, and the Vivaldi antenna can also be used as the feed for the beam steering [25,39]. The patch antenna was chosen because of its easy manufacturing and small transmission direction size, which shows the inherent advantage of the low-profile property, but the patch antenna is often limited by the narrow bandwidth, in general, the −10 dB bandwidth of patch antenna is less than 10%. Another shortcoming of the patch antenna is that it is difficult to keep the symmetric radiation pattern in the azimuthal direction because the asymmetrical radiation will bring the distortion to the radiation when the TA or RA is feed by off-set source or in the situation of beam steering [18,19].

The horn antenna itself has the advantages of higher gain and wider bandwidth, so it can bring a higher gain enhancement and broader bandwidth for the TA antenna. Though the horn antenna often suffers from the bulky volume and larger size in the direction of wave propagation, it is easy to achieve the symmetric radiation pattern in the azimuthal direction. However, the conventional pyramid and conical horn antenna fail to perform the symmetric pattern due to the fundamental mode excitation, like the Potter horn or corrugated horn, higher-order mode is introduced into the cavity [15,19,20,27,28,38,40]. In this paper, we utilize the advantages of Potter horn antenna to optimize a feed source at the frequency range of 26–30 GHz with symmetrical radiation pattern for the SLL reduction in full azimuthal directions. 

**Figure 1:**
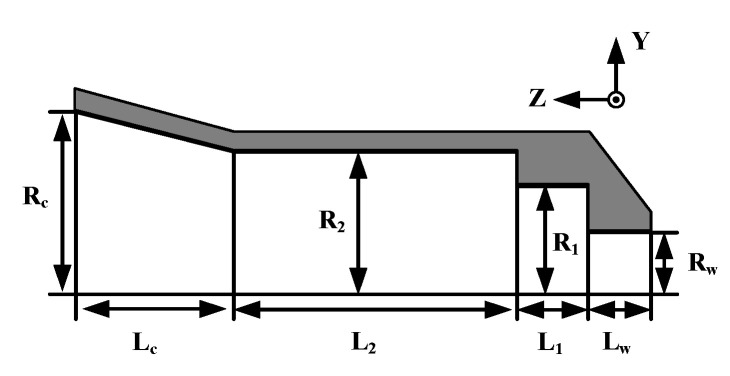
The cross-sectional view of the Potter horn antenna as the feed source.

**Figure 2:**
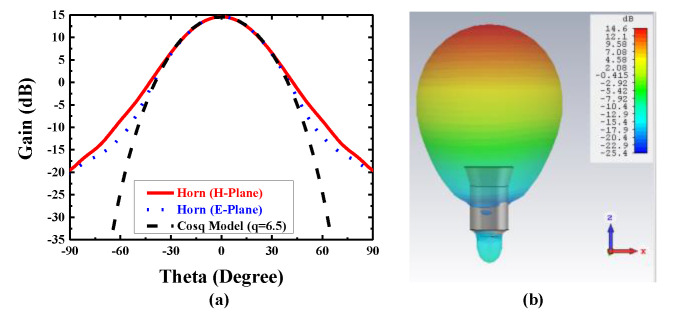
The radiation pattern of the proposed antenna. (**a**) Comparison of cos^q^ (θ) curve and the radiation pattern of the horn in E-plane and H-plane. (**b**) The 3D radiation pattern at 28 GHz.

Figure 1 shows the cross-sectional view of the Potter horn antenna operating at 28 GHz. The feed structure is 21.16 mm × 21.16 mm × 27.96 mm as the effective volume, which consists of a circular waveguide, two conjunctions, and a flared section. The horn is made of copper with a thickness of 1 mm. The gain is 14.6 dBi at 28 GHz, and half power beam width (HPBW) is 36° at E-plane and H-plane in order to simplify the further optimization about the radiation and the aperture efficiency. In Ref. [37], the horn antenna can be molded as the function of the cos^q^ (θ) in the scalar expression of far-field radiation pattern for a planar antenna array, and it is reasonable that q = 6.5. The curve of cos^q^ (θ) and the radiation pattern of the Potter horn antenna in E-plane and H-plane are shown in Figure 2. After optimization, it is obvious that the radiations not only in E-plane but also in H-plane match well with the theoretical curve of cos^q^ (θ) in the theta range from −45° to 45°. 

**Figure 3:**
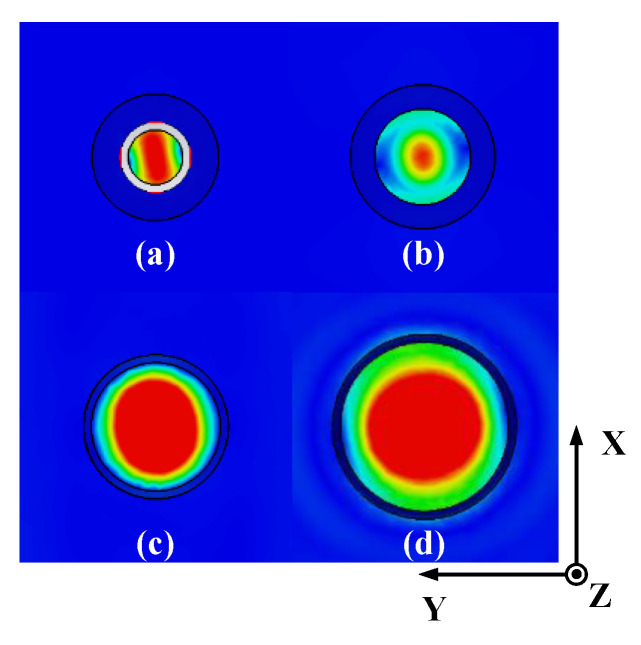
The electric field magnitude at the cross sections inside the optimized horn antenna: (**a**) inside the circular waveguide, (**b**) at the first abrupt change part, (**c**) at the second abrupt change part, and (**d**) at the flared aperture.

The values of the final optimized parameters are: *L*_w_ = 3.2 mm, *L*_1_ = 4.05 mm, *L*_2_ = 16.14 mm, *L*_c_ = 8.9 mm, *R*_w_ = 3.39 mm, *R*_1_ = 5.99 mm, *R*_2_ = 8 mm, and *R*_c_ = 10.58 mm. All the results are simulated by CST Microwave Studio. *L*_w_ is the length of the circular waveguide and the waveguide excites the dominant TE_11_ mode and generate a directive beam with asymmetric radiation pattern. *L*_1_ and *L*_2_ are the length of the two abrupt changes of the waveguide in which the first junction keeps the power in the TE_11_ mode, the second junction excites the TM_11_ mode, and meanwhile, the flared horn is worked to avoid the propagation of TM_12_ mode. The simulation result of return loss (S11) with a −10 dB bandwidth of the feed source is 14%, which is from 26.39 to 30.37 GHz. The side lobe levels are −26.2 dB and the Half Power Beam Width (HPBW) are about 36° in both the E-plane and the H-plane. As shown in Figure 3, the electric fields at the cross sections of the optimized Potter horn are presented. It is obvious that the symmetric aperture fields are generated at the horn’s cross section which lead to the symmetric radiation patterns regardless of the azimuthal angles.

### 2.2. Design of Unit Cell of MS

**Figure 4:**
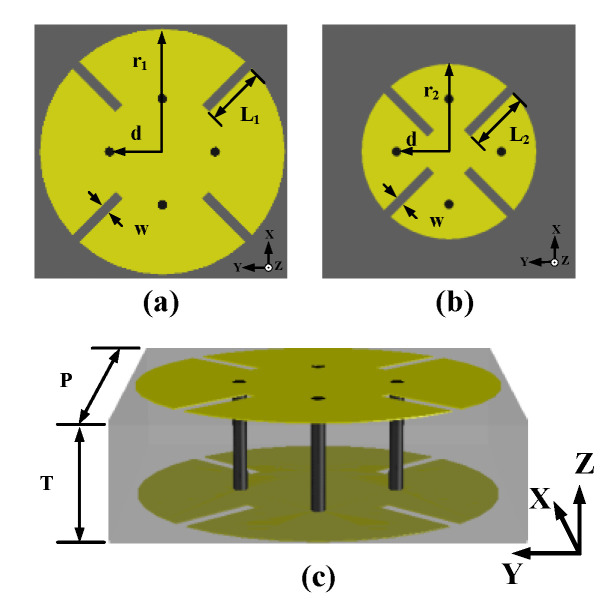
The structure of unit cell: (**a**) Unit Cell A, (**b**) Unit Cell B, and (**c**) side view of unit cell.

The proposed unit cells with two identical conductive layers printed on the both sides of the substrate and the slots control the changing of transmission coefficients; the whole structure is presented in Figure 4. The material of substrate is Rogers 4003C, which has the dielectric constant (ε_r_) of 3.38 and loss tangent of 0.0027. To get the phase and amplitude of transmission coefficient (S_21_), CST Microwave Studio is used to simulate the unit cell structure with period boundary condition in X and Y direction and the excitation of the normal plane wave in Z direction. In Figure 4c, the side length of square substrate (*P*) is 5.3 mm, the thickness (*T*) of substrate is 1.524 mm, and four vias are inserted in the substrate to connect the upper and bottom metallic layers.

The unit cells can be classified into two group: Unit Cell A as shown in Figure 4a and Unit Cell B as shown in Figure 4b. For Unit Cell A, *r*_1_ = 2.55 mm, *L*_1_ changes from 1. 28 to 1.85 mm, and a phase shift range from 360° to 156° is covered. For Unit Cell B, *r*_2_ = 1.83 mm, *L*_2_ varies from 0.61 to 1.29 mm, while the phase shifts from 156° to 0°. The residual parameters like the position of vias (*d*) and the width of slots (*w*) are kept the same in both Unit Cell A and Unit Cell B: *d* = 1.1 mm and *w* = 0.2 mm. Each unit cell has four vias at the symmetric positions away from the center. Only two different sizes of circular metallic patches are used to build the TA, and meanwhile, the length of slot is changed to get different transmission phase, which will be used to focus the beam. Unlike in the Ref. [28], the 304° phase shift is achieved by changing the side length of square patch and the position of via is varied with the patch size proportionally; the TA in this paper can cover the 360° phase shift with sweeping the length of slots on Unit Cell A and B, correspondingly. What’s more, the positions of via do not change with the dimension of patches, which means the positions of via are fixed on the array, and those will reduce the cost and the difficulty in the procedures of design and fabrication.

**Figure 5:**
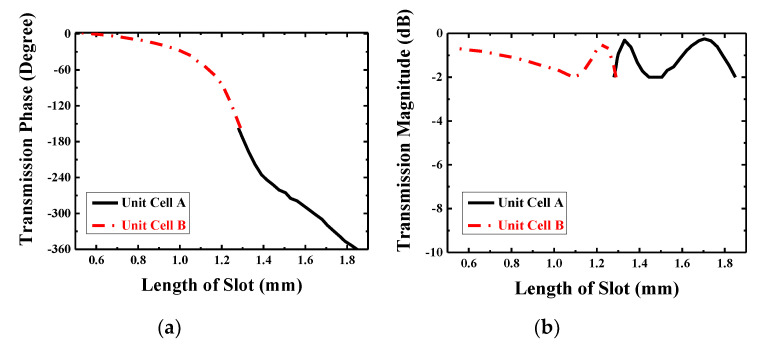
Transmission coefficients: (**a**) transmission phase and (**b**) transmission magnitude.

Figure 5 shows that the transmission coefficients change with the length of slots while other parameters remain unchanged in this process. Apparently, in Figure 5a, there is a seamless connection between the phase shift of Unit Cell A and Unit Cell B and the full 360° can be covered with a gentle slope to realize the requirement of focusing effect of transmission wave. The transmission magnitudes are all over −2 dB, which is proposed in Figure 5b, and for the high gain of TA design, design with less transmission loss is desirable; it is also be helpful in the optimization of SLL reduction in the following research of TA antenna.

The length of slots is varied from 1.28 to 1.85 mm with a 0.03 mm step in Unit Cell A, whereas in Unit Cell B, it is varied from 0.61 to 1.29 mm with a step of 0.02 mm, thus the relationship between the length of slots and the transmitted phase will be built for Unit Cell A and Unit Cell B, separately. In this way, it will be easier to confirm the specific length of slot at different positions on the array according to the phase distribution, which will be discussed in the next part.

### 2.3. Array of MS

**Figure 6:**
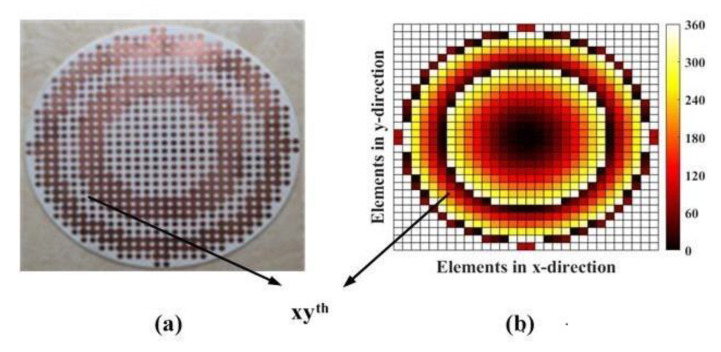
(**a**) The top view of the fabricated transmitarray (TA), and (**b**) the phase distribution of TA calculated by Equation (1).

The whole circular TA shown in Figure 6a is established by 660 elements with a diameter of 160 mm (*D* = 160 mm), and from the plot, we can figure out the approximate distribution of Unit Cell A and B. This is because that Unit Cell A covers the phase shift from 0° to 156° and Unit Cell B covers from 156° to 360°, and the related phase at the center of TA is 0°, so all elements are near the center based on Unit Cell B and away from the center of array; the elements based on Unit Cell A and those based on Unit Cell B will appear one after another. After confirming whether Unit Cell A or Unit Cell B is to be used for every element on the array, the corresponding relationship in Figure 5a is used to determine the length of slot for each element, so the final TA will be presented as the structure in Figure 6a. In this process, the required phase *φ*_xy_ at the position of *xy^th^* element on the array is based on (1) [37]:

(1) $$\varphi_{xy}=k(R_{xy}-\overset{\wedge }{r_{0}}\cdot \vec{r_{xy}})+\varphi_{0}$$

where *k* is the wave number, *R_xy_* is the distance between the feed position and the center position of *xy*th element on the array, $\hat{r_{0}}$ is the main beam direction, $\vec{r_{xy}}$ is the position vector of *xy*th element, and $\varphi_{0}$ represents the constant phase at the central point of the TA. Equation (1) is worked to calculate all the detailed phase values on the array as showed in Figure 6b. With the approximate functions of the element’s excitation, the radiation pattern can be simplified into the scalar form [24]:

(2) $$RP(\theta ,\varphi )=\sum_{x=1}^{X}\sum_{y=1}^{Y}\frac{\cos^{q}(\theta_{f}(x,y))}{\left|\vec{r_{xy}}-\vec{r_{f}}\right|}\cdot e^{jk(\vec{r_{xy}}\cdot \overset{\wedge }{\mu }-\left|\vec{r_{xy}}-\vec{r_{f}}\right|)}\cdot e^{j\varphi_{xy}}$$

(3) $$RP_{n}(\theta ,\varphi )=20\times \log_{10}(\frac{\left|RP(\theta ,\varphi )\right|}{\max(\left|RP(\theta ,\varphi )\right|)})$$

where *θ* is in the range of −90° to 90° and *φ* is in the range of 0–360°, *θ_f_* (*x*, *y*) is the spherical angle in the feed’s coordinate system, *q* is the feed pattern factor, which is used in the design of horn antenna with the value of 6.5, $\vec{r_{f}}$ is the position vector of the feed, and $\hat{\mu }$ is the observation direction. Based on (2), the normalized radiation pattern $RP_{n}\left(\theta ,\varphi \right)$ can be achieved and the main beam has a beam width from −10° to 10° with the SLL of −19 dB.

The element at the center of TA is selected carefully. It is true that $\varphi_{0}$ can be an theoretically arbitrary value because the focusing effect of the TA is based on the related phase distribution, which means that the related phase at the center being 0° will make the calculation of phase distribution easier so the phase at other positions will change based on the related 0° to imitate the function of optical lens. However, in this process, the transmission efficiency is regarded as 1, which means no loss is taken into consideration. However, in the actual situation, the design of the TA has to face the problem of transmission loss inevitably and this is always a research direction for the TA. What’s more, in the design of TA, there are mainly three parts of approximations needed to be paid attention. First, the transmitted phase and amplitude values obtained from the cell analysis in Figure 5 are in the case of normal incident plane wave, but in fact, elements in the array except the central one are illuminated by different degrees of oblique incident, so there is some deviation. Second, full 360° phase shifting is needed to achieve the focusing effect of the TA, as ideally, the theory requests continuous varying of phase. However, the elements on the TA are all block structures with the same volume, and we just regard the elements as volumetric free and put the center-point coordinate of every element on the array into the (1) to calculate the required phase and use those phases to build the model, so the phase values are presented in the form of cubes in Figure 6b. As a result of which, the phase shifting will be discontinuous, some phase errors will be caused inevitably. Third, the adjacent elements on the array are different from those around them and there is a feed source in the TA antenna so the mutual coupling are complex. However, in the analysis of unit cell, the data are acquired under the situation of periodic boundary condition with the plane wave excited. In general, we ignore the impacts of oblique incidence, discontinuity, and actual mutual coupling to design the TA.

**Figure 7:**
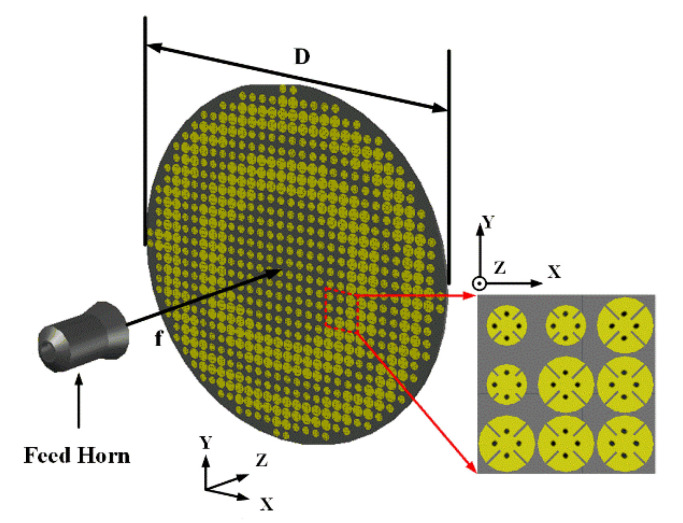
The simulation model of final TA and source antenna.

After the optimization of unit cell, we can keep all the transmission coefficient in the level of −2 dB, which is shown in Figure 5b. From that plot, it is obvious that the most part in Unit Cell B has a higher and smoother tendency changing with the length of slots than the magnitude tendency of Unit Cell A. Additionally, according to the first and second approximation discussed before, the farther from the center, the greater the design error may occur. Hence, we should put the elements with less transmission loss around the center as much as possible. In this situation, the Unit Cell B with the length of 1.29 mm (*L*_2_ = 1.29 mm) is selected as the four identical elements in the center because of the symmetrical distribution of elements. Other elements are determined based on the relationship between the length of slots and the related phase in Figure 5a; therefore, the distribution of elements with different dimensional information is presented in Figure 6a. Figure 7 presents the positional relation between TA and the feed horn. As described above, *D* is the diameter of TA, which has the value of 160 mm, and *f* is the distance from the center of source antenna to the center of TA, and the ratio between *f* and *D* (*f/D*) is an important criterion to judge the SLL and low-profile of a TA antenna. The smaller value of *f/D*, the better low-profile performance of a TA antenna and the worse of the backward radiation, so there is a tradeoff needed to be made.

**Figure 8:**
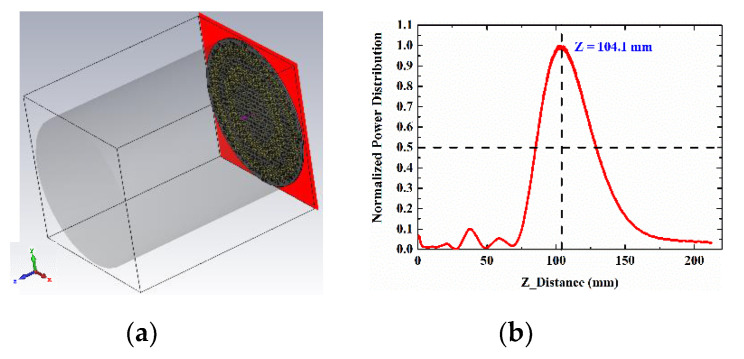
(**a**) TA with plane wave and (**b**) the normalized power distribution along intersection line of E-plane and H-plane.

**Figure 9:**
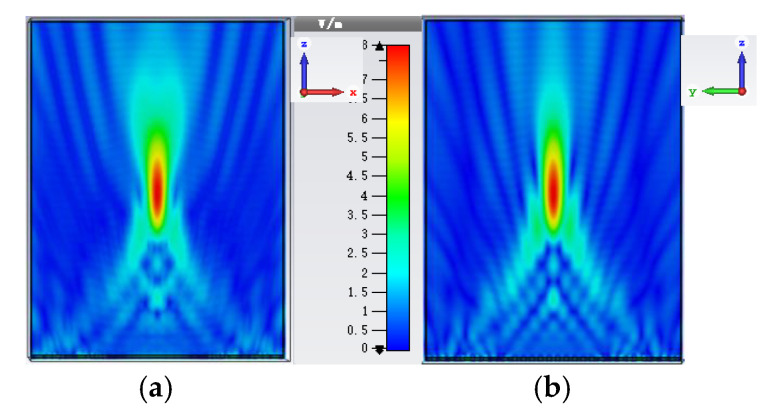
Electric field (Ex) amplitude distribution: (**a**) E-plane and (**b**) H-plane.

**Figure 10:**
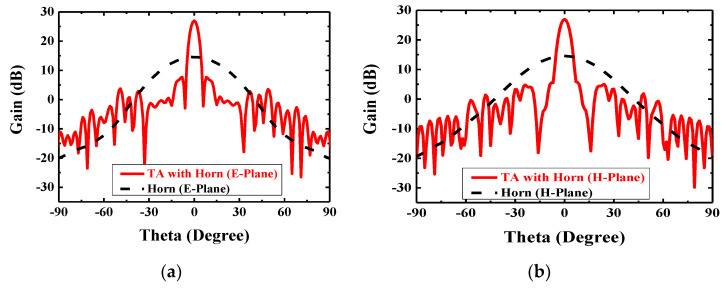
Simulated far-field radiation pattern of feed source and TA antenna in two cut planes at 28 GHz: (**a**) E-plane comparison and (**b**) H-plane comparison.

For the working principle, the TA is aimed to imitate the function of lens and the feed source is placed at the virtual focal point. When the quasi-spherical wave emitted by the feed crosses over the TA, it will compensate the phases and form a uniform wave front similar to a plane wave. Reversely, when the TA is illuminated by the plane wave, the wave will gather at one point theoretically just like the optical reversibility in the Fermat’s principle. In this way of analysis, a plane wave is placed at one transmission side of the TA like shown in Figure 8a, and the power density on the E-plane and H-plane of the other TA side will be shown as in Figure 9. Obviously, the energy is focused at an area, and this area is the approximate position to place the feed. The normalized energy distribution along the intersection line of E-plane and H-plane is proposed in Figure 8b. The peak in Figure 8b is the theoretical optimum of the feed position (*f*) which has a value of 104.1 mm, so the theoretical ratio of *f/D* will be around 0.65, and slight changing near 0.65 will be needed in the simulation to find the best position of feed. The simulation result is shown in Figure 10, in which the far-field gain of TA antenna is 27 dB at 28 GHz, SLL in E-plane is −19.6 dB and in H-plane is −21.7 dB, and compared with the radiation pattern of horn, the focusing effect of TA is significant in two cut planes.

### 2.4. Optimization Result

As the simulation of TA antenna established before, it has a gain of 27 dB with the SLL of −20 dB at 28 GHz, and this result is based on the optimized *f/D* of 0.65, but the position of feed is not the only part that can be optimized in a system of TA antenna. In order to reduce SLL or form multibeams in the desired direction, the phase and magnitude distribution of array can also be optimized [36].

In the Refs. [38,39], various parameters and conditions about the PSO were discussed and compared. In this paper, a 660-dimensional hyperspace is chosen as one possible solution to this phase optimization with 660 elements on the array. In this hyperspace, there are 15 particles with 5000 iterations flying around to find the best position separately (*p_best_*), and meanwhile, they share the information with others dynamically (*g_best_*), hence they have the tendency to be in the best position, which is the best one for all the particles. This swarm behavior is based on the Equations (4) and (5) of velocity updating and position updating:

(4) $$\begin{array}{l} v_{n+1}=\omega \times v_{n}+c_{1}\times rand()\times (P_{best,n}-x_{n})\\ +c_{2}\times rand()\times (g_{best,n}-x_{n}) \end{array}$$

(5) $$x_{n+1}=x_{n}+v_{n}\times t$$

where $\nu_{n}$ and $x_{n}$ are the particle’s velocity and the coordinate in the nth dimension, respective; *ω* is the inertial factor that relates to the previous direction of velocity, and it decreases from 0.9 to 0.4 linearly, which means that it has weaker and weaker relationship with the previous position and turns to the local best position at that moment. *C*_1_ and *C*_2_ are the leaning factor in which *C*_1_ is the self-learning factor and *C*_2_ is the global learning factors, and they are set as the same value of 2.0 to balance autonomy and group learning ability [38,39]. The function of random number rand (1) give a stochastic value between 0.0 and 1.0 in every iteration so that the particle can explore as largest possible hyperspace. Equation (5) shows how the particle move to the next position with the common time step of 1.0. The aim of this PSO is to optimize the phase distribution on the array so that the TA will have a SLL lower than −30 dB. The detailed process of PSO is presented below. First, the radiation pattern in the optimization process is regarded as (6) and (7):

(6) $$RP_{1}(\theta ,\varphi )=\sum_{x=1}^{X}\sum_{y=1}^{Y}\frac{\cos^{q}(\theta_{f}(x,y))}{\left|\vec{r_{xy}}-\vec{r_{f}}\right|}\cdot e^{jk(\vec{r_{xy}}\cdot \overset{\wedge }{\mu }-\left|\vec{r_{xy}}-\vec{r_{f}}\right|)}\cdot e^{j\varphi_{opt}}$$

(7) $$\varphi_{opt}=\varphi_{xy}+\varphi_{fluc}$$

where *φ_opt_* is the phase distribution, which needs to optimize. It contains two parts, one is the normal phase distribution based on (1), whereas another is the fluctuations added at the corresponding phase, and their sum are used in (6) to get the optimized radiation pattern in every iteration for all particles. The normalized radiation pattern in the optimization is Equation (8):

(8) $$RP_{1n}(\theta ,\varphi )=20\times \log_{10}(\frac{\left|RP_{1}(\theta ,\varphi )\right|}{\max(\left|RP_{1}(\theta ,\varphi )\right|)})$$

The ideal goal of the optimization has not related to *φ* and shown in Equation (9):

(9) $$RP_{goal}(\theta ,\varphi )=\{\begin{array}{cc} 0 & -10^{\circ }\leq \theta \leq 10^{\circ }\\ -30dB & 10^{\circ }\leq \left|\theta \right|\leq 90^{\circ } \end{array}$$

Then, the fitness function can be built based on the difference between *RP*_1*n*_ and *RP_goal_*, which is shown in Equation (10):

(10) $$fitness=\{\begin{array}{cc} \sum_{-90^{\circ }}^{90^{\circ }}\sum_{0^{\circ }}^{360^{\circ }}\left(RP_{1n}-RP_{goal}\right) & RP_{1n}>RP_{goal}\\ 0 & else \end{array}$$

The value of this fitness function is called as the Cost of the optimization, and the lower cost, the closer to the goal of −30 dB SLL. Theoretically, when the Cost equals to zero in the iterations, the optimization finishes completely.

But in fact, in most cases of optimization, when the Cost is small as an acceptable value and the Cost no longer changes in the process of iterations, the PSO is regarded as finished. In this paper, for 15 particles with 5000 times of iterations for each of them, the final Cost has a value of 101.2 and the Cost has hardly changed at the last 1000 iterations. Generally, the values of *φ_fluc_* is the optimized phase fluctuations, and the TA will be established based on the phase distribution of *φ_opt_*. The final simulation will use this optimized structure to achieve high gain and low SLL, which will be presented in the next section.

## 3. Result and Discussion

The simulated far-field radiation pattern in E-plane and H-plane of the feed antenna and the TA before optimization at 28 GHz is shown in Figure 10, the gain increases from 14.6 to 27.1 dB. As shown in Figure 10a, the HPBW reduces from 36° to 5.0° in E-plane and from 36° to 5.4° in H-plane as shown in Figure 10b.

**Figure 11:**
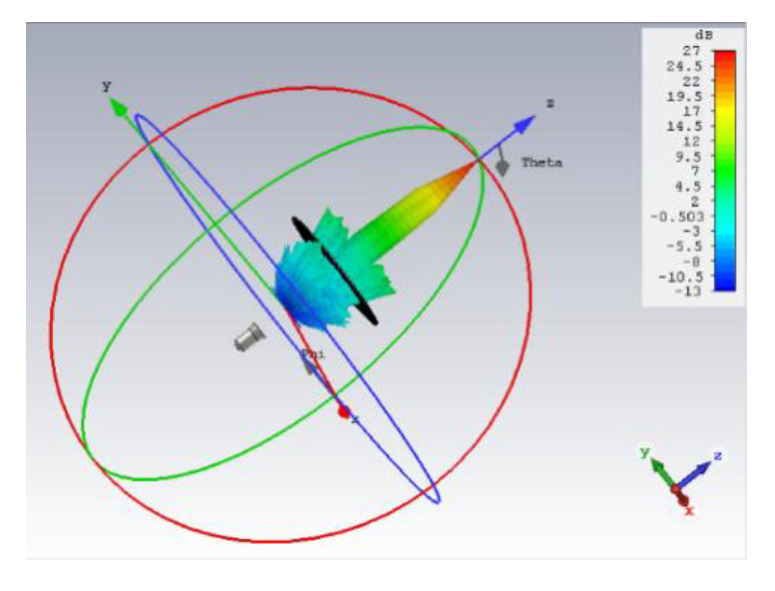
A 3D radiation pattern of the optimized TA antenna at 28 GHz.

**Figure 12:**
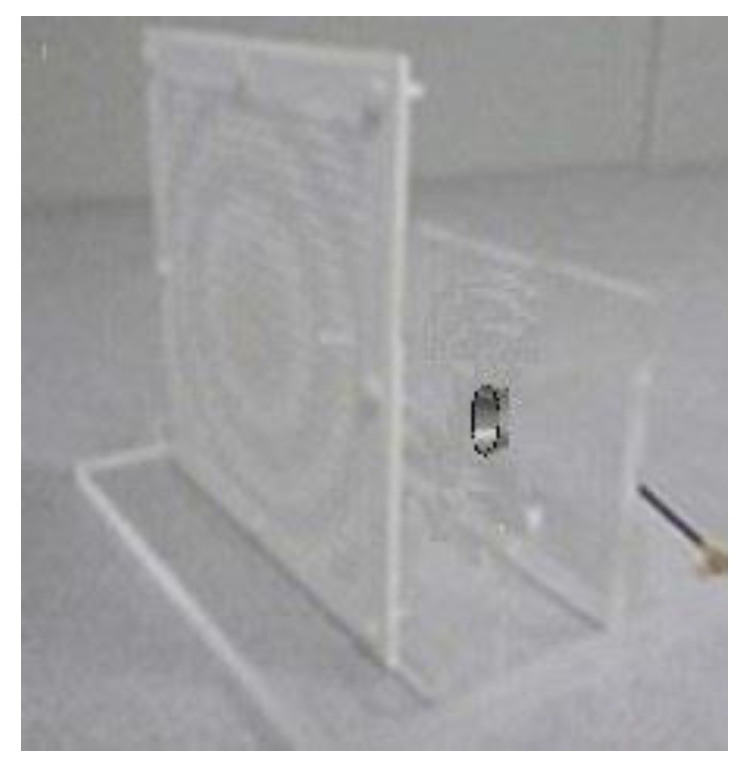
Photograph of experimental setup of the proposed antenna.

Figure 11 demonstrates the 3D simulated radiation pattern of TA antenna at 28 GHz, which indicates that a pencil-shaped beam is achieved when the quasi-spherical wave emitted by the feed horn passes through the optimized TA with a low SLL. A good performance of focusing effect is visible, and the beam points clearly to the z positive direction. The experimental setup of the proposed antenna can be seen in Figure 12. The whole TA occupies an area of 160 mm × 160 mm × 140.1 mm with a *f/D* value of 0.65. Because of the symmetry of the structure, the parameters of unit cell of the TA can be obtained by one radial row. The optical parameters for two kinds of unit cells of first line along horizontal direction from center to edge are *L*_11_ = 1.27 mm, *L*_12_ = 1.29 mm, *L*_13_ = 1.26 mm, *L*_14_ = 1.27 mm, *L*_15_ = 1.16 mm, *L*_16_ = 1.02 mm, *L*_21_ = 1.81 mm, *L*_22_ = 1.70 mm, *L*_23_ = 1.36 mm, *L*_17_ = 1.27 mm, *L*_18_ = 0.95 mm, *L*_24_ = 1.80 mm, *L*_25_ = 1.47 mm, *L*_26_ = 1.27 mm, and *L*_19_ = 1.12 mm, and $L_{i}(i=1,2)$ refers to Unit Cell A and Unit Cell B, respectively.

**Figure 13:**
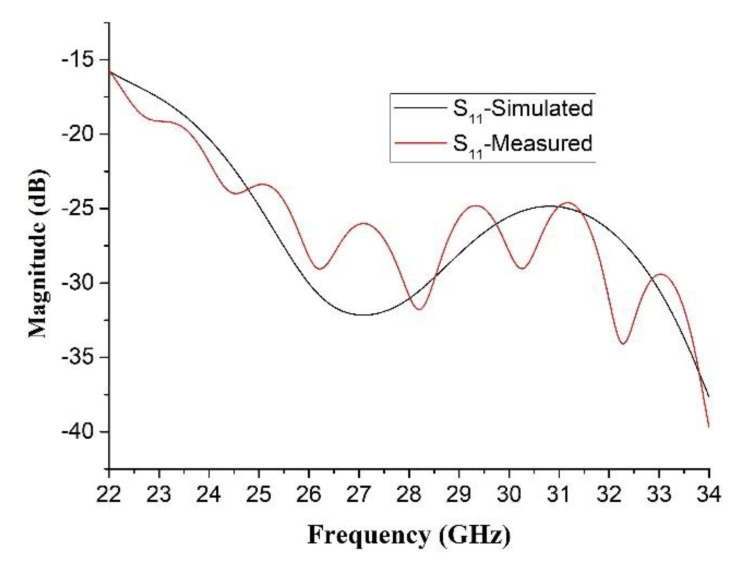
Simulated and measured S11 of the proposed antenna.

**Figure 14:**
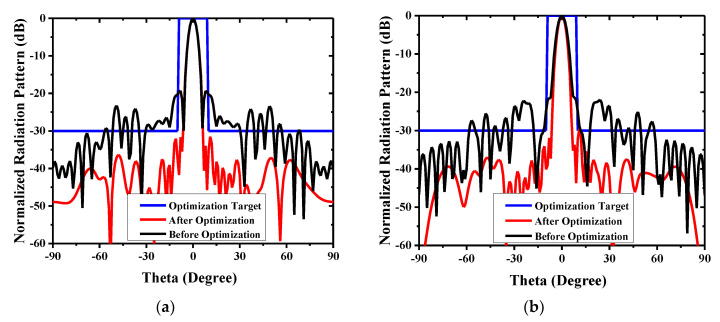
Comparison of the radiation pattern in two cut planes at 28 GHz: (**a**) E-plane comparison and (**b**) H-plane comparison.

**Figure 15:**
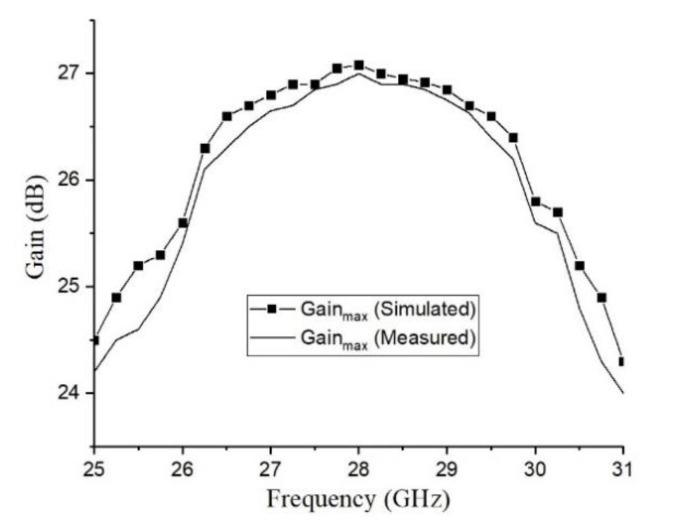
Simulated and measured maximum gains within the operating frequency band.

The reflection coefficient of the proposed antenna is shown in Figure 13. The feed antenna has wide passband, and the Gain the whole antenna is limited by TA. Figure 14 indicates the compared radiation pattern of measured results with the simulated results before the optimization in two cut planes, and it is easy to find that the SLL in E plane changes from −19.6 to −30 dB and in H plane from −21.7 to −30 dB, and all the side lobes, except the first side lobe, are kept below −35 dB. In summary, the SLL of this TA antenna achieves the level of −30 dB from the level of −20 dB by using the PSO. Figure 15 illuminates the measured gains within the operating frequency band. The PSO is good for the design of directional array antenna, which has great potentials in the design of beam steering antenna and MIMO (Multi Input Multi Output, MIMO) array antennas. Besides the analysis of gain and SLL, the property of aperture efficiency is also an aspect to evaluate the performance of TA antenna. The method to enhance the aperture efficiency is also a research direction, which is related to the f/D, spillover radiation, and edge scattering. Generally, the aperture efficiency is based on (11) and (12).

(11) $$e_{ap}=\frac{10^{G/10}}{D_{\max}}$$

(12) $$D_{\max}=\frac{4\pi \cdot A}{\lambda_{0}^{2}}$$

where *e_ap_* is the aperture efficiency, *D*_max_ is the directivity in the main beam (unit: dimensionless), *G* is the maximum gain (unit: dB), the physical area of TA is marked as *A* (unit: m^2^), $\lambda_{0}$ is the wavelength at the desired central frequency point (unit: m^2^), which is 28 GHz in this paper. Then (12) will be substituted into (11), so we can get the aperture efficiency of the optimized TA antenna at *K*a band, which shows 23% in simulation. Finally, it is obvious that the TA antenna can achieve a performance of high gain, low SLL and high aperture efficiency with the optimized feed horn and the optimized phase distribution using PSO. As shown in Table 1, the TA antenna has higher gain, wider bandwidth and higher aperture efficiency at the central frequency of 28 GHz.

## 4. Conclusions

This paper presents a SLL reduced TA antenna based on the two-layered transmitarray (TA), and the whole TA occupies an area of 160 mm × 160 mm × 140.1 mm with a *f/D* value of 0.65. The two-layer structure uses the mutual coupling of vias to accumulate the transmission phase without additional substrate layers. The optimized horn antenna with a cos^q^ (θ) mode simplifies the radiation-pattern calculation of the TA. Finally, the PSO is applied to optimize the SLL from −20 to −30 dB, which achieves a peak gain of 27 dBi at 28 GHz, 11.8% 3 dB gain bandwidth, and 23% aperture efficiency. This optimized TA could also be excited by the offset feed or MIMO antenna as the function of beam forming or beam steering in the field of 5G communication and radar system. The proposed TA antenna will play great prospects in the further research, especially when the high gain, low SLL and wideband is needed.

## Figures and Tables

**Table 1:** Comparison of the optimized TA antenna with some published array antenna.

Ref.	Freq. (GHz)	TA Thickness $\left(\lambda_{0}\right)$	Array Size $\left(\lambda_{0}^{2}\right)$	SLL (dB)	Number of Elements	Bandwidth	Gain_max_	Aperture Efficiency (%)
[25]	28	0.18	89.5	−18	484	11.%	24.2	24.5
[28]	20	10.5	368.5	−22.5	2032	10.2%	33.0	40
[38]	13.5	0.76	164.7	−22	621	9.8%	29.95	50
This paper	28	0.14	173.3	−30	660	11.2%	27	28
